# Role of Lectin-Like Oxidized Low Density Lipoprotein-1 in Fetoplacental Vascular Dysfunction in Preeclampsia

**DOI:** 10.1155/2014/353616

**Published:** 2014-07-06

**Authors:** Felipe A. Zuniga, Valeska Ormazabal, Nicolas Gutierrez, Valeria Aguilera, Claudia Radojkovic, Carlos Veas, Carlos Escudero, Liliana Lamperti, Claudio Aguayo

**Affiliations:** ^1^Department of Clinical Biochemistry and Immunology, Faculty of Pharmacy, University of Concepción, 4070386 Concepcion, Chile; ^2^Department of Basic Science, Faculty of Medicine, Universidad Católica de la Santísima Concepción, 4090541 Concepcion, Chile; ^3^Vascular Physiology Laboratory, Group of Investigation in Tumor Angiogenesis (GIANT), Group of Research and Innovation in Vascular Health (GRIVAS Health), Department of Basic Sciences, Faculty of Sciences, Universidad del Bío-Bío, 4081112 Chillán, Chile

## Abstract

The bioavailability of nitric oxide (NO) represents a key marker in vascular health. A decrease in NO induces a pathological condition denominated endothelial dysfunction, syndrome observed in different pathologies, such as obesity, diabetes, kidney disease, cardiovascular disease, and preeclampsia (PE). PE is one of the major risks for maternal death and fetal loss. Recent studies suggest that the placenta of pregnant women with PE express high levels of lectin-like oxidized LDL receptor-1 (LOX-1), which induces endothelial dysfunction by increasing reactive oxygen species (ROS) and decreasing intracellular NO. Besides LOX-1 activation induces changes in migration and apoptosis of syncytiotrophoblast cells. However, the role of this receptor in placental tissue is still unknown. In this review we will describes the physiological roles of LOX-1 in normal placenta development and the potential involvement of this receptor in the pathophysiology of PE.

## 1. Introduction 

Preeclampsia is a leading cause of maternal and neonatal morbidity and mortality. Despite the fact that this disease has been studied for more than hundreds of years, pathophysiology is still unclear. However, there is no doubt that one of the underling mechanisms associated with occurrence of preeclampsia is the alteration in the endothelial function, a phenomenon described as endothelial dysfunction. In turn, endothelial dysfunction is associated with unbalance between generation and activity of free radicals, including nitric oxide (NO) and superoxide anion (O_2_^−^), in favor of generation of nitrative and/or oxidative stress. Among several mechanisms related to generation of oxidative stress during preeclampsia, recent evidences suggest that expression of LOX-1, a scavenger receptor for oxidized low density lipoprotein (oxLDL), may be a keystone receptor that needs to be investigated, since it is involved in many processes related to pathophysiology of preeclampsia. Thus, the aim of this review is to describe the physiological and pathophysiological roles of LOX-1 in normal and preeclamptic pregnancies.

## 2. Vascular Endothelial Function and Nitric Oxide Generation

The endothelium is a monolayer of cells located in the inner wall of blood vessels and is the first physical and metabolic barrier between blood and tissues. The endothelium is involved in the regulation of hemodynamic function in physiological state, a phenomenon associated with synthesis and release of vasoactive molecules including nitric oxide (NO), prostaglandins, and thromboxanes [1]. In physiological conditions there is a tight balance between the generation of these different agents, and any disturbance in this equilibrium generates a pathological condition denominated endothelial dysfunction. In general, endothelial dysfunction is a syndrome characterized by loss in antithrombotic, angiogenic, and inflammatory and vasodilator function. This syndrome has been observed in different pathologies, such as obesity, diabetes, kidney disease, cardiovascular disease, and preeclampsia [2–6]. Endothelial dysfunction is generally related to low bioactivity or bioavailability of NO, which in turn is associated with reduced vasodilator capacity and loss of vascular protection against harmful agents [7–9].

Nitric oxide is a potent vasodilator agent, inhibits platelet aggregation and leukocyte adhesion to the vascular wall, prevents proliferation of muscle cell, and reduces the expression of adhesion molecules and chemokines involved in monocyte infiltration [10]. Nitric oxide is derived from the conversion of L-arginine into L-citrulline (i.e., L-arginine/NO pathway) through a reaction catalyzed by NO synthase (NOS). There are at least three NOS isoenzymes coded by independent genes: neuronal (nNOS or NOS I, 12q24.2), inducible (iNOS or NOS II, 17cen-q11.2), and endothelial (eNOS or NOS III, 7q35-36) [11–13]. Bioavailability of NO is regulated by several mechanisms including reaction with reactive oxygen species (ROS) [14]. The interaction between NO and the superoxide anion (O_2_^−^) produces the relatively long-lived potent prooxidant peroxynitrite anion (ONOO^−^), which is highly toxic, initiates lipid peroxidation, and nitrates tyrosine residues on proteins, thus inhibiting or promoting signal transduction pathways [15]. NO also modulates mitochondrial respiration and the redox state of mammalian cells [16]; it could react with sulfide-containing molecules (such as albumin) to form nitrosothiol compounds [17] and promotes vascular endothelial insulin transport [18]. These evidences show us the important role of NO in regulating vascular function and that is why abnormalities in their synthesis lead to alterations in vasodilation and changes in vascular function.

In 1997, Sawamura et al. [19] successfully identified the major endothelial receptor for oxidized LOX (oxLDL), a lectin-like oxidized LDL receptor-1 (LOX-1). LOX-1 is a key molecule in the generation of endothelial dysfunction [20, 21]. LOX-1 activation is associated with cell proliferation, apoptosis, and cell migration [22, 23]. Binding of oxLDL to LOX-1 rapidly activates NADPH oxidase, resulting in rapid increase of intracellular reactive oxygen species (ROS), including O_2_^−^ and H_2_O_2_ [24], with concomitant decreased intracellular NO [25], and decline cytochrome P450 activity resulting in decrease of endothelium-derived hyperpolarizing factor [26] and endothelial cells dysfunction [27].

### 2.1. Receptor LOX-1

LOX-1 is an endothelial receptor for circulating oxLDL that has been studied extensively in pathological states, such as atherosclerosis, diabetes, coronary arterial heart disease, and hypertension [22, 23, 28]. LOX-1 is highly expressed in blood-vessel-abundant tissues such as placenta, lung, marrow, and spinal cord, is moderately expressed in hippocampi, testicle, and large arteries, and is slightly expressed in heart, skeleton muscle, and ovary [19]. At the cellular level, LOX-1 is expressed in macrophages, vascular smooth muscle cells, monocytes, and endothelial cells [27]. In vitro, the basal expression of LOX-1 is low, but the expression is highly induced by proinflammatory and prooxidative stimuli in endothelial cells, smooth muscle cells, and macrophages. The stimuli include TNF-*α* [29, 30], heparin-binding-EGF [31], oxLDL [32], oxidative stress [33], remnant-like lipoprotein particles (RLPs) [34], angiotensin II [35], D-glucose [36], and lysophosphatidylcholine [32].

Vascular LOX-1 gene expression is markedly enhanced in hypertensive rats [37–39], hyperlipidemic rabbits [40], and diabetic rats [28]. Ischemia reperfusion also increases the LOX-1 expression in myocardium and kidney [41–44]. Several clinical drugs can inhibit the expression of vascular LOX-1. These include antihypertensive (angiotensin II receptor agonist, calcium channel blockers, and angiotensin-converting enzyme inhibitors), antihyperlipidemics (statins) [45], antidiabetic (sulfonylurea, biguanide, and peroxisome proliferator-activated receptor-*γ*/PPAR*γ* agonist) [46], antithrombotic (aspirin) agents [47], and dihydrotestosterone [48].

Although LOX-1 was initially characterized as a receptor for oxidized LDL, just like other scavenger receptors, LOX-1 exhibits binding activity for multiple ligands. The precise oxLDL epitope recognized by LOX-1 is not known but is thought to be peptide based [49]. Several studies have shown that LOX-1 can recognize other modified lipoproteins including hypochlorite modified high-density lipoprotein [45], but not native LDL. LOX-1 also binds anionic polymers such as polyinosinic acid and carrageenan [49], anionic phospholipids including phosphatidylserine [50], apoptotic bodies [51], activated platelets [52], AGEs (advanced glycation end-products) [53], and both gram-positive and gram-negative bacteria [54].

The human LOX-1 gene (OLR1; low density lipoprotein oxidized receptor 1, OMIM no. 602601) localizes within natural killer-gene complex (NKC) as a single-copy gene and is assigned to the p12.3-p13.2 region on the short arm of human chromosome 12 [55]. OLR1 gene has more than 7000 bp and is composed of 6 exons separated by 5 introns; introns 1 to 5 have a length that is in the range of 102–246 bp while exon 6 is longer extending up to 1722 bp. Exon 1 encodes the 5′-UTR region and the cytoplasmic domain of LOX-1; exon 2 encodes the remaining portion of the cytoplasmic domain and the transmembrane domain; exon 3 encodes the neck domain; exons 4, 5, and 6 encode the C-type lectin domain and the 3′-UTR region of the protein [56]. The LOX-1 promoter is constitutively active only at low levels but may rise in different pathological conditions, including hypertension, hyperlipidemia, diabetes, and atherosclerosis [22], and its expression can be induced by different ligands or activators, including oxLDL, shear stress, phorbol 12-myristate 13-acetate (PMA), advanced glycation end products (AGEs), and others [32, 56, 57]. LOX-1 protein has a molecular weight of 50 kDa and belongs to the family of the C-type lectin [19]. It is synthesized as a precursor protein of 40 kDa, which undergoes subsequent four glycosylation sites found in the extracellular domain C-terminus, being finally processed to the mature form of 48 kDa [58, 59].

The existence of certain SNP gene OLR1 is associated with an increased risk in developing acute infarction (AMI). For instance, patients with allele T/T or C/T in the 3′-UTR region are at increased risk (OR 3.74) of developing AMI [60]. Another SNP related to cardiovascular disease is G501C polymorphism, which is found inversely proportional to the degree of stenosis and severity of coronary artery disease [61]. Furthermore, a polymorphism located in exon 4, which produces an amino acid change at position 167 (K167N) of the C-type lectin domain of the protein, causes a reduction in the binding and internalization of oxLDL [62, 63]. A total of 7 SNP in the OLR1 gene have been identified, six of them, located within introns 4, 5 and 3′-UTR, comprised a complete linkage disequilibrium block associated with the elevated risk for myocardial infarction [60]. Since SNPs are located in noncoding regions they do not produce changes in receptor expression; however, modulate the relative abundance of two transcripts generated by alternative splicing. One of these products corresponds to the entire shape of the receptor, while the other is a truncated version, called LOXIN.

LOXIN represents a variable isoform as a consequence of alternative splicing of LOX-1 receptor, which confers protection against proatherogenic effects, contributing to the formation of an inactive heterodimer with LOX-1 [64, 65]. LOXIN, lack of exon 5, lost 2/3 of the lectin-like domain and therefore is unable to bind oxLDL [64]. Interestingly, expression of LOXIN in COS-7 cells [64], endothelial cells, human endothelial progenitor cells (unpublished data), and mononuclear cells of peripheral blood [63, 65] results in a decrease in apoptosis mediated by oxLDL, revealing a potential cardioprotective effect of LOXIN. Currently, there are no studies that demonstrate the role of LOXIN in preeclampsia.

**Figure 1:**
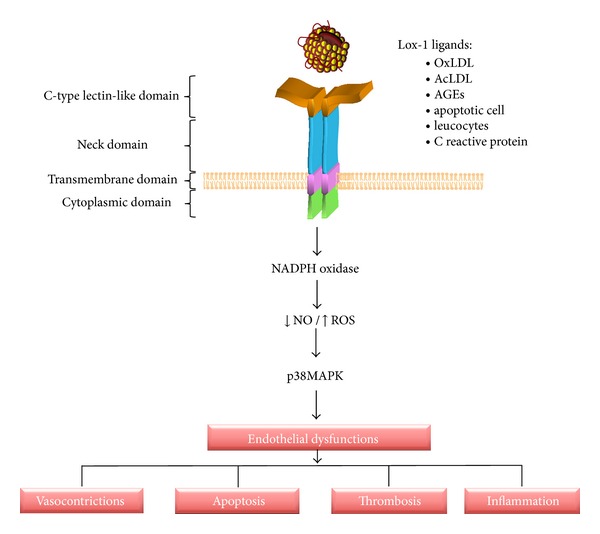
Structure and LOX-1 signaling. LOX-1 has four domains: cytosolic domain (1–34 amino acids), transmembrane domain (amino acids 35–61), neck domain (amino acids 62–143), and CTLD (amino acids 144–263). The LOX-1 activation by different ligand increases phosphorylation of p42/44MAPK and p38MAPK and the expression of gp91phox subunit of NADPH oxidase, causing an increase in reactive oxygen species and a decrease in NO and hence endothelial dysfunction manifested by cell apoptosis, thrombosis, inflammation, and vasoconstriction.

Through ROS generation, LOX-1 stimulates gene expression by activating two signal transduction pathways involving either p38MAPK or ERK1/2 and PI3K, both causing NF-*κ*B activation [22, 23]. NF-*κ*B regulates expression of vascular genes including P-selectin, VCAM-1, ICAM-1, MCP-1, and M-CFS, involved in the attachment and activation of monocytes [66–68]. Decrease of eNOS and Bcl-2 and increase of matrix metalloproteases (MMP1, 3, 9) and Fas expression cause cells injury and apoptosis of endothelial cells [69]. LOX-1 activation can also lead to cell proliferation that is blocked with anti-LOX-1 neutralizing antibody. Recent evidence has shown that a complex formed by LOX-1 and membrane type 1 matrix metalloproteinase (MT1-MMP) plays a crucial role in RhoA and Rac1 activation signaling pathways in Ox-LDL stimulation. Blockade of LOX-1 or MT1-MMP inhibits cell invasion, endothelial NO synthase protein downregulation, RhoA-dependent and NADPH oxidase activity, and reactive oxygen species generation, mediated by Rac-1 [70]. All these evidences suggest the close relationship between NO bioavailability and ROS generation during LOX-1 activation in human endothelial cells (Figure 1).

### 2.2. LOX-1 and Placenta

The placenta is the regulator of nutrient composition and supply from mother to fetus and the source of hormonal signals that affect maternal and fetal metabolism; appropriate development of the placenta is crucial to normal fetal development [71]. During pregnancy, cholesterol is an essential component for placental and fetal development, used by the placenta for the synthesis of steroid hormones [72]. Physiological adaptations of maternal lipoprotein metabolism occur throughout pregnancy, leading to an increase in lipoprotein concentrations from second trimester to term in preparation for the catabolic phase of late pregnancy, a period of rapid fetal growth.

The placenta exhibits a high expression of LOX-1 mRNA [19] and the maternal lipid profile is associated with placental protein expression of OLR1 [73] which suggest a crucial role of this receptor in the placental function. For instance, it has been suggested that LOX-1 might be involved in trophoblast invasion in early pregnancy [74, 75] and accelerated trophoblast apoptosis and endothelial dysfunction preeclampsia [76]. In addition, using a trophoblastic cell line, the choriocarcinoma JAR, it has been reported that LOX-1 is responsible for 40–50% of oxLDL uptake [77]. Developmental studies looking for expression of LOX-1 in murine and human placentas have described higher expression of LOX-1 during early to midgestational stages than late gestation [78]. In murine placenta, LOX-1-expressing cells were fibroblast-like stromal cells in metrial glands and decidua basalis and trophoblast cells in the junctional and labyrinth zones. In the human placenta, LOX-1 was detected in villous cytotrophoblasts in first trimester and term placentas. Other studies show that LOX-1 is localized in extravillous trophoblasts of first trimester placentas [74, 79] and in syncytiotrophoblast of normal and preeclamptic term placentas, the latter being higher than normal [76].

Despite the fact that LOX-1 is present in placenta, it is intriguing what would be its functions. From studies using LOX-1 deficient mice it is known that LOX-1 is not a lethal gene, since animals are fertile [80] and had no detectable abnormalities during pregnancy. However, cells that express LOX-1 in the placental or in the maternal-placental interphase may be involved in management and evolution of oxidative stress or inflammatory response during pregnancy. In this regard, studies of Satoh et al. [78] demonstrated that LOX-1 is localized in fibroblast-like cells, a cell type that is closely associated with uterine NK cells; then this receptor might be involved in regulation of trophoblast invasion and maternal vascular remodeling during implantation.

## 3. Preeclampsia Overview

Preeclampsia is a major cause of maternal and infant morbidity and mortality worldwide [81–83]. Stillbirth is more common in preeclamptic pregnancies while one-third of infants of preeclamptic women are growth restricted [84, 85]. The incidence of preeclampsia is variable, affects 7 to 10% of all pregnancies [81, 85–88], and depends on the demographic and sociocultural characteristics of the population, as well as the criteria used for diagnosis of the disease. According to the criteria of the International Society of the Study of Hypertension in Pregnancy, the pregnancy-induced hypertension is defined as “*diastolic blood pressure >90 mm Hg occurring after week 20 of gestation with proteinuria (either 300 mg protein per day or a urinary protein/creatinine ratio 30 mg/mmol)”* [89]. However, proteinuria is no longer an absolute requirement for the diagnosis of preeclampsia according to the 2013 guide of The American College of Obstetricians and Gynecologists [90]. The removal of proteinuria as a diagnostic requirement for preeclampsia/eclampsia reflects the recognition that many women (14%) with preeclampsia do not have proteinuria [91], and such women have historically experienced delays in diagnosis and treatment as a result. Alternatively, the diagnosis may be established by the presence of hypertension associated with thrombocytopenia (platelet count less than 100.000/*μ*L), impaired liver functions (elevated blood concentrations of liver transaminases to twice the normal concentration), development of renal insufficiency (serum creatinine concentration greater than 1.1 mg/dL or a doubling of the serum creatinine concentration in the absence of other renal diseases), pulmonary edema, or new-onset cerebral or visual disturbances [92, 93].

The risk factor of developing preeclampsia is higher in women with diabetes, thrombophilia, and obesity with either preexisting vascular disease or conditions associated with increased cardiovascular risk, including renal disease, hypertension, and with previous preeclampsia [94]. Clinical manifestations are highly variable and may occur antepartum, intrapartum, or postpartum [95]. The preeclampsia can lead to problems in the liver, kidneys, brain, and the clotting system. Moreover, as stated above, preeclampsia is associated with low birthweight and perinatal deaths due to premature birth and intrauterine growth restriction [81, 96].

Although etiology of preeclampsia is still unknown, there is no doubt that this condition is associated with endothelial dysfunction, which leads to occurrence of classical clinical syndrome of hypertension, proteinuria, and edema [83, 97–100]. It is believed that the placenta has a key role in the pathophysiology of preeclampsia since the symptoms and complication of preeclampsia disappear after delivery of placenta [100, 101]. On the other hand, the uterus or fetus is probably not necessary for the development of this pathology, since there have been reports of preeclampsia with abdominal pregnancies [102] or hydatidiform moles [103].

**Figure 2:**
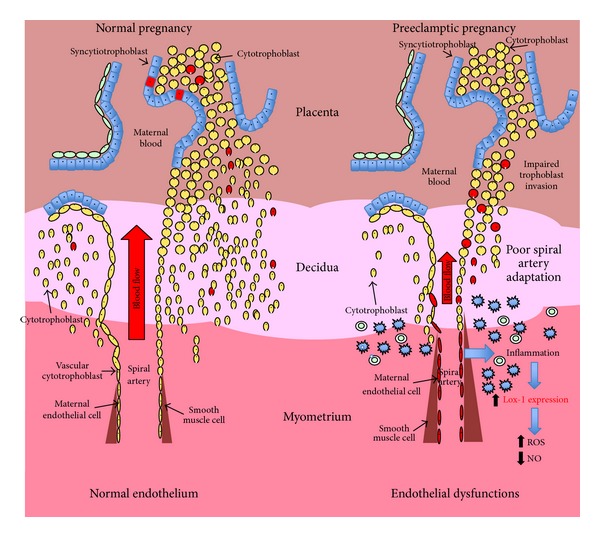
Preeclampsia and LOX-1. In a normal pregnancy adequate trophoblast invasion produces spiral arteries in saccular without a muscular layer, whereby the placental bed is a set of high flow and low resistance. However, in preeclampsia the uteroplacental blood flow was reduced due to an incomplete trophoblast invasion that generates a high strength and low blood flow. Under this condition, placentation arterial lesions were subsequently produced inducing an inflammatory condition, thus increasing perfusion deficit and oxidative stress. In normal pregnancy, lower levels of LOX-1 were found expressed in cells that are part of the trophoblast and syncytiotrophoblast. On the other hand, in preeclampsia LOX-1 levels increase significantly, which may be responsible for the generation of high levels of ROS and decreased levels of NO.

Although endothelial dysfunction is well described in preeclampsia, the underling mechanism behind this alteration is not completely understood. Several studies have described the release of harmful molecules that may cause endothelial damage from the placenta toward maternal circulation during this disease [104–110]. For instance, the release of soluble receptor type 1 of vascular endothelial growth factor (sFlt-1) [88, 111], soluble endoglin [112], and lipid peroxides [113] has been described. Nowadays, many groups believe that trophoblastic microparticles/nanoparticles shedding toward maternal circulation are a keystone event in the generation of preeclampsia. The investigation is focused on trying to understand how these particles are generated, what would be their content, and what they are doing in the maternal physiology. Actual beliefs explain that incomplete or absent trophoblastic invasion to the uterine spiral arteries is related to reduced uteroplacental blood flow and placental ischemia [83, 105, 114, 115] (see Figure 2). In this scenario, hypoxia/reoxygenation of placental tissue leads to synthesis of free radicals [116] and in particular synthesis and release of reactive oxygen species (ROS), such as superoxide anion, which easily reach the maternal circulation directly or via modification of macromolecules where it can react with NO leading to reduction of NO bioavailability and impaired vascular response [117].

On the other hand, early placental development is characterised by rapid cell differentiation and migration, matrix remodelling, and angiogenesis. The enzyme NADPH oxidase is a major source of superoxide anions implicated in signalling pathways regulating these processes [118]. Normal pregnancy has an increased oxygen requirement by different organs, including the fetoplacental unit [119]; therefore it is necessary to maintain a tight control of the levels of oxidants and antioxidants during pregnancy. Diverse studies comparing biomarkers of oxidative stress in normal pregnant women and nonpregnant control subjects have shown that lipid peroxidation is enhanced during the second trimester of pregnancy as well as late in gestation [120], which is associated with decreased activity levels of SOD and GPX and decreased glutathione [121, 122]. The placenta is an important generator of lipid peroxides [120] but is also rich in antioxidant defense elements such as SOD, catalase, GPx, glutathione, and vitamins C and E [123]. ROS play important roles in normal placental development and may also play a role in influencing the growth trajectory of the placenta, and its component cell types, in contrast to the oxidative stress considered responsible for the pathophysiology of many diseases pathologies of pregnancy [124].

Growing evidence suggests that placental oxidative stress is involved in the etiopathogenesis of preeclampsia. Besides this, preeclampsia is characterized by a diminished antioxidant capacity [125]. Women with preeclampsia show increased biomarkers of oxygen radical damage and impairment of antioxidant defence. Placental tissue shows higher level of markers of lipid peroxidation such as F2-isoprostanes [126], nitrotyrosine and 4-hydroxynonenal [127]. Moreover, the activities of SOD and GPX and the tissue levels of vitamin E were significantly lower in preeclamptic placentas compared to normal placentas, whereas the activity of catalase was significantly higher in preeclampsia. When analyzed at the mRNA level, expression of SOD and GPX was found significantly lower in preeclamptic versus normal placentas, but there was no change in the catalase expression [128]. In other studies, the tissue levels of endogenous antioxidant proteins such as superoxide dismutase, glutathione peroxidase, thioredoxin reductase, and thioredoxin were all reduced in preeclamptic tissue compared with normal tissue [129]. Glutathione is a major endogenous water-soluble antioxidant, and women with preeclampsia had lower glutathione concentrations in plasma [130] and in erythrocytes compared with normotensive control women [131]. Moreover, significantly lowered levels of vitamins E and C were observed in preeclamptic women as compared with controls [132]. Overall, the evidence suggests that increased oxidative stress and reduction in antioxidant defense mechanisms may contribute to the disease process in preeclampsia.

### 3.1. LOX-1 and Preeclampsia

Maternal hyperlipidemia is one of the striking changes to take place in lipid metabolism during even normal pregnancy [133]; however elevated maternal circulating cholesterol is a risk factor leading to fetal endothelial dysfunction, which could have serious consequences to the growing fetus [134]. There are several indirect evidences that suggest the participation of LOX-1 in the pathophysiology of preeclampsia. Thus, small dense LDL is also increased in the plasma of women with preeclampsia and LDL particles are more susceptible to oxidation, resulting in the generation of oxLDL [135]. Also it has been reported that levels of antibodies to oxLDL are elevated in women with established preeclampsia and in pregnant women with a history of repeated abortion [76]. Human placental cells express many lipoprotein receptors such as the LDL receptor (LDLR; [136]), the LDL receptor-related protein (LRP; [137]), the VLDL receptor (VLDL receptor; [138]), the scavenger receptors [139–141], and the LOX-1 [142].

More direct evidences have described high levels of LOX-1 in rat model of preeclampsia [143]. Moreover, the inhibition of LOX-1 prevents endothelial dysfunction in an in vitro model of preeclampsia [144]. On the other hand, it was found that upregulation of LOX-1 in HUVECs is exposed (24 h) to plasma from women with preeclampsia [135], a phenomenon that was also observed when plasma from preeclampsia was used in a rat model [145]. Indeed, in this animal model it has been demonstrated that exposure of plasma from women with preeclampsia with high content of oxLDL increased blood brain barrier permeability after acute exposition [145], suggesting that LOX-1 might be involved in occurrence of brain alterations associated with eclampsia. Other authors previously demonstrated an increase in the LOX-1 and arginase expressions in the maternal vasculature of women with preeclampsia when compared with normotensive pregnant women [135]. Contrasting to these results, but using placental tissue, Chigusa et al. [77] reported a decreased LOX-1 expression in preeclampsia.

Recently, Morton et al. [143] demonstrated in a rat model of preeclampsia a significant increase in the expression of both LOX-1 receptor and eNOS in the thoracic aorta, which was associated with increased superoxide anion generation and subsequent decreased endothelial function. LOX-1 activation has been shown to induce several intracellular signaling pathways, including increased expression of chemokines and adhesion molecules, triggering the CD40/CD40L pathway that activates the inflammatory cascade and increased generation of reactive oxygen species, such as superoxide anion in endothelial cells [146, 147]. Studying the soluble isoform of LOX-1 (sLOX-1), other groups have found no significant differences in sLOX-1 concentration in the plasma of patients with preeclampsia compared with matched control plasma [144].

Lee et al. [76] demonstrated that in vivo localization and the upregulated expression of LOX-1 are associated with reduced placental SOD-1 activity in preeclampsia, which fit perfectly in the well-described phenomenon of increased oxidative stress and reduced antioxidative defense in the preeclamptic placenta. Similar evidence suggests that oxLDL and LOX-1 could activate the NADPH oxidase enzyme system to generate superoxide anion. Sankaralingam et al. [135] demonstrated increased NADPH oxidase activity in cultured endothelial cells in response only to plasma from women with preeclampsia, which was significantly reduced by blocking with anti-LOX-1 or siRNA to LOX-1. Then, all these results suggest that LOX-1 is involved in the pathophysiology of preeclampsia.

## 4. Concluding Remark

LOX-1 is an important scavenger receptor of Ox-LDL and plays an important role in the pathogenesis of atherosclerosis. Ox-LDL causes endothelial dysfunction and its accumulation is the first step in the development of cardiovascular disease. LOX-1 activation via Ox-LDL is thought to be involved in the initiation and the development of other different pathological conditions, including hypertension, hyperlipidemia, diabetes, and preeclampsia.

The underlying participation in the initial development of normal placenta and the pathophysiology of preeclampsia seem far more complex than originally thought as LOX-1 plays a fundamental role in the process of trophoblast differentiation and syncytiotrophoblast formation. The activation of LOX-1 in early pregnancy contributes to inducing migration and invasion of the trophoblast and cell apoptosis, where this phenomenon seems to depend on the formation of reactive oxygen species. However, during preeclampsia there is an increased expression of LOX-1 receptor, suggesting that this receptor may contribute to the endothelial dysfunction observed in this pathology. The increased expression and activity of LOX-1 in preeclampsia would favor the formation of free radicals that alter cellular function. Free radicals have opposite effects depending on their concentration; high expression of LOX-1 in preeclampsia may be associated with high levels of ROS and endothelial dysfunction, whereas low levels of ROS promote cell proliferation and migration.

The evidence shown in this review suggests the important role of LOX-1 in normal and pathological conditions, and their identification could be useful for understanding the development of preeclampsia. However, more information is needed in order to better understand the role of LOX-1 in the development of the placenta and in some pregnancy complications, such as preeclampsia.
